# *Mycobacterium tuberculosis* Infection-Driven Foamy Macrophages and Their Implications in Tuberculosis Control as Targets for Host-Directed Therapy

**DOI:** 10.3389/fimmu.2020.00910

**Published:** 2020-05-12

**Authors:** Dahee Shim, Hagyu Kim, Sung Jae Shin

**Affiliations:** ^1^Department of Microbiology, Institute for Immunology and Immunological Diseases, Brain Korea 21 Program for Leading Universities and Students (PLUS) Project for Medical Science, Yonsei University College of Medicine, Seoul, South Korea; ^2^Department of Life Science, Research Institute for Natural Sciences, College of Natural Sciences, Hanyang University, Seoul, South Korea

**Keywords:** *Mycobacterium tuberculosis*, foamy macrophage, tuberculosis, immune responses, lipid metabolism, lung inflammation, host-directed therapy

## Abstract

Tuberculosis (TB) is a leading cause of death worldwide following infection with *Mycobacterium tuberculosis* (Mtb), with 1.5 million deaths from this disease reported in 2018. Once the bacilli are inhaled, alveolar and interstitial macrophages become infected with Mtb and differentiate into lipid-laden foamy macrophages leading to lung inflammation. Thus, the presence of lipid-laden foamy macrophages is the hallmark of TB granuloma; these Mtb-infected foamy macrophages are the major niche for Mtb survival. The fate of TB pathogenesis is likely determined by the altered function of Mtb-infected macrophages, which initiate and mediate TB-related lung inflammation. As Mtb-infected foamy macrophages play central roles in the pathogenesis of Mtb, they may be important in the development of host-directed therapy against TB. Here, we summarize and discuss the current understanding of the alterations in alveolar and interstitial macrophages in the regulation of Mtb infection-induced immune responses. Metabolic reprogramming of lipid-laden foamy macrophages following Mtb infection or virulence factors are also summarized. Furthermore, we review the therapeutic interventions targeting immune responses and metabolic pathways, from *in vitro, in vivo*, and clinical studies. This review will further our understanding of the Mtb-infected foamy macrophages, which are both the major Mtb niche and therapeutic targets against TB.

## Introduction

Tuberculosis (TB) is a chronic inflammatory disease caused by a *Mycobacterium tuberculosis* (Mtb) infection (1). When the Mtb bacilli become inhaled into alveoli, the bacilli are phagocytosed by alveolar macrophages in the lung (2). Phagocytosed Mtb uses various approaches to avoid host defense mechanisms, such as inhibition of phagosome maturation, expression of virulence-associated factors, inhibition of phagolysosomal fusion, and protection from reactive oxidative radicals (3, 4). Following infection with Mtb, alveolar macrophages migrate to the interstitium and induce inflammatory responses, resulting in the extravasation of dendritic cells, neutrophils, natural killer cells, T cells, and B cells (2). These infiltrated immune cells surround the infected alveolar macrophages, which are reservoirs of Mtb, to construct TB granulomas (5–7). Thus, understanding the fate of alveolar macrophages at the initial infectious phase is critical for preventing TB pathogenesis.

During the construction of TB granulomas, Mtb-infected macrophages accumulate lipid bodies in their cytosolic area, differentiating into foamy macrophages, which are hallmarks of TB lesions (8, 9). The accumulated bubble-like lipid bodies contain cholesteryl esters and triglycerides (10). Mtb-infected foamy macrophages play central roles in granuloma development, maintenance, and infection dissemination (9). In advanced granulomas, the core region is characterized by caseous necrosis, which further leads to the formation of a lipid-rich environment (6, 8, 9, 11). In granulomas, Mtb can grow and persist in foamy macrophages and the necrotic core (7). When foamy macrophages leave the original granuloma, a secondary granuloma is established, promoting dissemination (11). As the primary niche for Mtb, determining the features of Mtb-infected foamy macrophages is essential for investigating and controlling TB pathogenesis. This review describes the current understanding of foamy macrophages infected with Mtb.

## Macrophages

### Differentiation and General Features of Lung Macrophages

Macrophages are well-classified by their ontogeny (12–14). The functions and phenotypes of macrophages are influenced by their developmental origins and locations. Macrophages are generally formed as three major precursors: embryonic yolk sac precursor, fetal liver precursors, and bone-marrow derived blood monocytes (13, 14). In the steady state lungs, macrophages consist of alveolar macrophages and interstitial macrophages (15). The alveolar macrophages originate from fetal liver macrophages and fetal monocytes under the control of granulocyte/macrophage colony-stimulating factor (GM-CSF), peroxisome proliferator-activated receptors-gamma (PPAR-γ), and the lung microenvironment (16–19). To maintain homeostatic regulation, alveolar macrophages have a unique enhancer repertoire, including *Spi-C* and *Car4*, which are induced by the macrophage lineage-determining factor PU.1 in the lung-specific microenvironment (20, 21). A recent study demonstrated that mammalian target of rapamycin (mTOR) signaling is also required for the self-renewing ability of alveolar macrophages, accompanied by a distinctive metabolic signature based on the expression of sterol regulatory binding protein (SREBP) target genes (22). While undergoing tissue imprinting in the lungs, alveolar macrophages generally express immunosuppressive genes, including tumor growth factor-β (TGF-β) and interleukin-10 (IL-10) (17, 23). Alveolar macrophages have unique phenotypic features; they are loosely adherent, round-shaped cells expressing high levels of cluster of differentiation 206 (CD206), which detects microbial carbohydrates and high levels of scavenger receptors, such as macrophage scavenger receptor class A and macrophage receptor with collagenous structure (23). Notably, alveolar macrophages also express high levels of CD11c and CD170; moreover, their expression of F4/80 and CR3 is low or absent compared with that in other parts of the lung or peripheral tissue-resident macrophages (17).

In contrast, interstitial macrophages originate from primitive yolk sac macrophages and bone marrow monocytes (15). These cells are smaller and have monocyte-like morphology, which is characterized by a high nuclear/cytoplasm ratio with cytosolic vacuoles compared to alveolar macrophages (24). Interstitial macrophages express high levels of CD11b, CD64, F4/80, and the proto-oncogene tyrosine-protein kinase MER (25). Following invasion by microorganisms, interstitial macrophages play a role in the second-line defense via their phagocytosis and antigen presentation abilities (26). These interstitial macrophages have diverse immune responses depending on the activation stimulus, explained by the concepts of M1 and M2 macrophages. The activated spectrums and concepts for the M1 and M2 macrophages are further described below.

### General Functions and Metabolic Programing of M1 and M2 Macrophages and Their Distinct Roles in Lung Inflammation by Mtb Infection

Overall, when macrophages are infected with bacteria or viruses, they elicit pro-inflammatory responses by releasing anti-microbial proteins and cytokines, such as complement proteins, tumor necrosis factor α, IL-1β, IL-6, IL-12, and IL-23 (27–29). These pro-inflammatory macrophages can activate endothelial cells in the blood vessels to support extravasation of other immune cells into inflamed areas (28, 29). Macrophages also trigger T cell responses through their antigen presenting abilities via major histocompatibility complex II molecules (30). *In vitro*, macrophages can be classically activated by lipopolysaccharide or interferon γ (IFN-γ) to mimic bacterial infection or pro-inflammatory activation, respectively. These classically activated macrophages are named as M1 macrophages (31). From the perspective of immunometabolism, M1 macrophages are well-known to drive pro-inflammatory responses during the metabolic switch for glycolysis with a broken tricarboxylic acid (TCA) cycle (32, 33). The pentose phosphate pathway and NAD^+^ salvage pathway are essential for generation of mitochondrial reactive oxygen species (ROS), which induces DNA damage following M1 macrophage activation (34). In addition to massive glycolysis with an impaired TCA cycle, glucose-derived carbons are incorporated into fatty acids or sterol via lipogenesis in activated M1 macrophages (35).

Macrophages also play pro-resolving or anti-inflammatory roles that depend on signal transducer and activator of transcription-6 (STAT-6) and IL-10 release (27–29). These macrophages are involved in the phagocytosis of apoptotic cells, induction of collagen deposition, and coordination of tissue integrity to reinforce tissue repair, regeneration, and fibrosis (27, 36). There is also a method to polarize anti-inflammatory macrophages through the activation of IL-4/IL-13 or IL-10 *in vitro*; these alternatively activated macrophages are generally named as M2 macrophages (31). M2 macrophages preferentially utilize oxidative phosphorylation and fatty acid oxidation (FAO) to drive anti-inflammatory responses (37). In M2 macrophage activation with IL-4 treatment, mitochondrial metabolism is regulated by polyamine biosynthesis to regulate the integrity of the TCA cycle and electron transport chain (38). Collectively, M1 and M2 macrophages may undergo distinct lipid metabolic reprogramming, followed by their differential immune responses.

**Figure 1:**
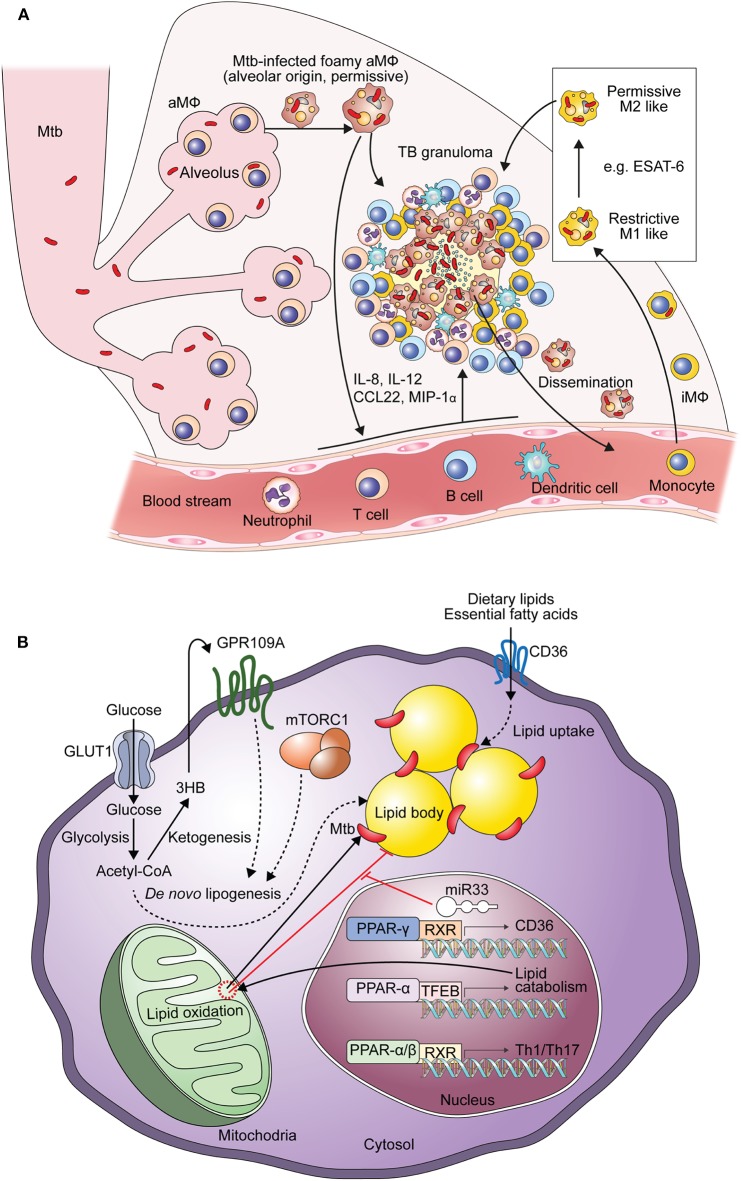
Generation of Mtb-infected foamy macrophages during the formation of TB granulomas. **(A)** Composition of Mtb-infected foamy macrophages during TB pathogenesis. Alveolar macrophages initially infected by Mtb and translocated into the interstitial space to generate immune responses. With the extravasation of immune cells Mtb-infected alveolar macrophages differentiate into foamy macrophages. Infiltrated interstitial macrophages are also infected with Mtb and further differentiate into foamy macrophages. In the early stage of Mtb infection, macrophages show pro-inflammatory responses like M1 macrophages contributing to the restriction of Mtb survival. ESAT-6, a representative virulence factor of Mtb, polarizes these M1 macrophages into M2 macrophages to induce permissive responses in Mtb survival in the chronic stage of TB. These Mtb-infected foamy macrophages are hallmarks of TB granulomas; translocation of Mtb-infected foamy macrophages induces dissemination of Mtb. “aMΦ” and “iMΦ” indicate “alveolar macrophage” and “interstitial macrophage,” respectively. **(B)** Metabolic perturbation by Mtb infection to generate foamy macrophages with Mtb infection, lipid accumulation leads to the generation foamy macrophages via metabolic reprogramming. In the early stage of Mtb infection, excessive glycolysis with defective mitochondrial respiration contributes to *de novo* lipogenesis. Acetyl-CoA, a product of glycolysis, is metabolized to 3-hydroxybutyrate (3-HB) by ketogenesis to induce GPR109A signaling. *De novo* lipogenesis is also induced by signal transduction of GPR109A and mTORC1 signaling, which is induced by macrophage activation. Nuclear receptors, such as those in the PPAR and LXR family, also contribute to both metabolic reprogramming and immune responses. The expression of miR33 is induced by Mtb and miR33 inhibits lipid catabolism, supporting Mtb survival. Direct and indirect processes are indicated by arrows and dotted arrows, respectively.

When infected with Mtb, both alveolar and interstitial macrophages play important roles in defending against TB and modulate immune responses (39). Mtb-infected alveolar macrophages exhibit enrichment in gene sets such as those involved in lipid uptake, oxidative phosphorylation and fatty acid oxidation, similar to M2 macrophages (39, 40). Mtb-infected alveolar macrophages express an antioxidant transcriptional signature via NRF2-dependent pathways, resulting in impaired control of Mtb growth with reduced inflammatory responses in the early stage of infection (40, 41). Mtb enhances the expression of genes in alveolar macrophages that are involved in Mtb division, growth, ribosomal protein synthesis, cell wall synthesis, fatty acid import, mycolic acid biosynthesis, the TCA cycle, and β-oxidation (40). Because the features of interstitial macrophages are determined by immunological stimuli, TB lesions have been investigated using M1 and M2 macrophages to dissect the immune responses occurring during TB progression (42, 43). In the early stage of Mtb infection, interstitial macrophages are typically differentiated to M1 macrophages. Mtb-infected interstitial macrophages express gene sets for cell adhesion, chemotaxis, ROS biosynthesis, nuclear factor-κB responses, hypoxia, and glycolysis *in vivo* (39, 40). In M1-like interstitial macrophages, Mtb shows a gene signature related to the response to environmental stresses and a non-replicative state (40). Mtb-infected M1 macrophages are transformed into M2 macrophages over time by the 6-kDa early secretory antigenic target (ESAT-6), which is a major virulence factor of Mtb (43). In addition to controlling the bacterial burden, modulating granuloma formation, and immune responses, Mtb-infected macrophages contribute to TB dissemination (44, 45). Mtb was shown to translocate from the phagolysosome to the cytosol, thereby eliciting host cell apoptosis in an ESAT-6–dependent manner (44). Apoptotic cells are then phagocytosed by newly infiltrating macrophages to generate the primary granuloma, and Mtb-infected macrophages egress to the distal tissues, contributing to the initiation of secondary granuloma formation (45) (Figure 1A).

From the perspective of immunometabolism, the Warburg effect in M1 macrophages generates pro-inflammatory responses, such as the secretion of IL-1β, which is known as a beneficial cytokine against Mtb (46, 47). Induced Warburg effects in the early phase of Mtb infection also generate ROS with the activation of hypoxia inducible factor 1α, which is necessary to induce Mtb specific IFN-γ dependent immunity (48, 49). IL-12, another cytokine secreted by M1 macrophages, can generate protective immune responses against Mtb (50). In contrast, M2 macrophages possess less bactericidal activities compared to M1 macrophages against Mtb infection (42). IL-10, a representative cytokine secreted from M2 macrophages, suppresses antimycobacterial immunity and promotes Mtb survival (51). It has been reported that another M2 macrophage-secreted cytokine, TGF-β, suppresses IFN-γ responses from T cells against Mtb in the lungs of mice and humans infected with TB; deletion of TGF-β signaling decreases the bacterial burden via the generation of cytotoxic T cell responses in TB granulomas (52).

Collectively, these investigations suggest the dynamic activation status of Mtb-infected macrophages (Figure 1A). Alveolar macrophages show enhanced lipid metabolism via PPAR-γ and inflammatory properties that facilitate infection and persistence of Mtb bacilli (18). In the progression of TB granulomas, blood monocyte-derived macrophages infiltrate and polarize to M1 macrophages. In the early phase of Mtb infection, excessive glycolysis with increasing lipids drives fatty acid synthesis in M1 macrophages. Further differentiation to M2 macrophages by ESAT-6 is closely linked to FAO with anti-inflammatory responses, providing favorable environments for Mtb survival (43). Therefore, these lipid metabolic reprogramming pathways may be considered targets for supporting host-directed therapy (HDT) to elicit anti-TB immune responses.

## Interaction Between Mtb and Mtb–Driven Foamy Macrophages

### Characteristics of Mtb-Infected Foamy Macrophages and the Utilization of Their Lipids by Mtb

Alterations of metabolic pathways are involved not only in the inflammatory responses of macrophages but also in the transformation of Mtb-infected macrophages into foamy macrophages, which are the major contributors to TB pathogenesis (Figure 1B), as previously described (5, 6). Foamy macrophages are named based on their morphology as they contain bubble-like lipid bodies in their cytoplasm (53). Mtb-infected foamy macrophages have different features compared to non-foamy macrophages (Table 1, upper). Specifically, Mtb-infected foamy macrophages express higher levels of MHCII, CD11c, CD40, and CD205, similar to dendritic cells, but show reduced capacity for antigen processing (54). Moreover, Mtb-infected foamy macrophages induce nitric oxide with elevated secretion of TGF-β to suppress T cell responses (55–57).

**Table 1:** Comparison of general characteristics between foamy and non-foamy macrophages, and metabolic interventions of Mtb-infected foamy macrophages for host-directed therapy.

**Cell type**	**Mtb states**	**Features**	**Surface marker**	**Immune responses**	**References**
Non-foamy macrophages	Replicative	Highly-phagocytic	CD11b^+^ CD64^+^ F4/80^+^ MertK^+^	Not reported	(25, 58)
Foamy macrophages	Dormant, non-replicative (after 6 days of infection)	Less-bactericidal Less-phagocytic	CD11b^+^CD11c^hi^ MHCII^hi^ CD40^hi^ CD205^hi^	Reduced antigen processing capacity	(54, 58)
				Suppressive effects on effector T cells via higher level of nitric oxide	(55, 56)
				Reduced TNF-α and IL-1α secretion Elevated TGF-β secretion	(57)
**Metabolic perturbation**	**Reported conditions**	**Experimental type**	**Mtb strain**	**Effect on TB control**	**References**
Glycolysis boosting	Treatment with metformin	Type 2 diabetes patients (cohort study)	NA^*^	Beneficial effects on prevention and treatment against TB	(88–90)
Glycolysis inhibition	Treatment with 2-deoxyglucose	BMDMs from C57BL/6	Erdman	Increased Mtb burdens	(39)
Increased lipid uptake via CD36	Genetic ablation of PPAR-γ using shRNA transfection	THP-1 cells with PPAR-γ knockdown	H37Ra, H37Rv	Decreased both Mtb burden and lipids	(71)
Increased lipid efflux by ATP-binding cassette transporter	Genetic ablation of LXR-α using shRNA transfection	THP-1 cells with LXR-α knockdown	H37Ra, H37Rv	Increased Mtb burden and intracellular lipids	(71)
*De novo* fatty acid synthesis inhibition	C75 treatment	THP-1 cells and human MDM	H37Rv	Lowering bacterial burden and lipid accumulation	(91)
*De novo* triacyglyceride synthesis inhibition	Rapamycin treatment for blocking mTORC1	Human MDM	H37Rv	Reduced both Mtb burden and lipid accumulation	(10)
	Everolimus and temsirolimus treatment for blocking mTORC1	Patients with metastatic renal cell carcinoma (cohort study)	NA^*^	Aggravation of TB progression by their immunosuppressive activities	(92, 93)
*De novo* cholesterol synthesis inhibition	Treatment with simvastatin in combination with rifampicin, pyrazinamide, and isoniazid	THP-1 cells and BALB/c mice	H37Rv	Beneficial effects on anti-TB therapy	(79)
	Treatment with simvastatin in combination with rifampicin, pyrazinamide, and isoniazid	J774 cells and BALB/c mice	CDC1551	Increased first-line anti-TB drug efficacy	(82)
	Treatment with atorvastatin	THP-1 cells and human MDM	H37Rv	Decreased both Mtb survival and intracellular lipids	(91)
	Treatment with seven different statins	Patients with metabolic syndrome (cohort study)	NA^*^	Lowering risk of active TB progression	(83)
	Treatment with statins in combination with anti-TB drugs	Patients with pulmonary TB (cohort study)	NA^*^	Not associated with improved outcomes of pulmonary TB	(81)
	Treatment with seven different statins in combination with anti-TB drugs or not	Type 2 diabetes patients (cohort study)	NA^*^	Not associated with decreased TB development	(80)
Fatty acid oxidation	Treatment of etomoxir, as CPT1a inhibitor	BMDMs from C57BL/6	Erdman	Decreased bacterial burdens	(39)

NA

* *, not applicable; BMDM, bone marrow-derived macrophage; CPT1a, carnitine palmitoyltransferase 1a; LXR-α, liver X receptor-alpha; MDM, monocyte-derived macrophage; Mtb, Mycobacterium tuberculosis; mTORC1, mammalian target of rapamycin complex 1; PPAR-γ, peroxisome proliferator-activated receptor-gamma; shRNA, short hairpin RNA; TB, tuberculosis*.

Upon infection, intracellular Mtb is located in the phagosomes, and the membrane of Mtb-containing phagosomes is enclosed and interacts with lipid bodies (58). A recent study demonstrated that the phagosome–lipid body interaction is regulated by the mycobacterial cell wall components lipoarabinomannan and phosphatidylinositol mannoside mediated by the late endosome marker Rab7 (59). After the phagosomes surrounded lipid bodies, Mtb translocated to lipid bodies for the utilization of lipids, such as cholesterols, fatty acids, and triglycerides, as carbon sources for survival. Mtb takes advantage of cholesterol and fatty acids in foamy macrophages to generate energy and metabolic intermediates via the expression of isocitrate lyases for the glyoxylate cycle (60). To utilize lipids, Mtb contains abundant genes encoding lipid transporters and lipolytic enzymes. It has been reported that LucA and Mce1 are expressed in Mtb to import fatty acids (61). Rv0200/OmamB, Rv0172/Mce1D, Rv0655/MceG, and Rv0966c are also known as fatty acid transporters of Mtb in foamy macrophages (62). Rv2672 is the membrane-associated Mtb protein mycobacterial secreted hydrolase 1 and is required for Mtb persistence via utilization of host lipids under hypoxic conditions (63). In addition to Mtb survival, fatty acids are required for the synthesis of virulence-associated lipids, including polyketide lipids phiocerol-dimycoseroic acid, poly-acylated trehaloses, sulfolipids, and mycolic acids (60). Increased accumulation of triglycerides and elevated levels of triglyceride synthetase 2 have been reported in the modern Mtb Beijing strain compared to the ancient Mtb Beijing strain. Moreover, these elevated levels of triglyceride are associated with rapid disease development (64).

Furthermore, it has been suggested the use of lipids in host cells is related to dormancy of Mtb (58, 65–67). It is reported that Mtb persists in a dormant non-replicative state in foamy macrophages compared to infect non-foamy macrophages (58). Mtb utilize fatty acids from host cells to generate intracellular lipid inclusions, which are lipid bodies in the cytoplasm of mycobacteria. Bacilli with these inclusions show persistently arrested growth in response to stress (65, 66). Particularly, utilization of host triglycerides is required to obtain the dormancy-like phenotypes of Mtb in foamy macrophages (68). The Mtb mutant with reduced triglyceride synthesis is more sensitive to antibiotics compared to wild-type Mtb (65). The region of difference 1 protein in Mtb contributes to increasing the levels of intracellular triglycerides in Mtb by enhancing the expression of diacylglycerol *O*-acyltransferase, a key enzyme in triglyceride synthesis (67). Therefore, reducing lipid bodies in Mtb-infected foamy macrophages may control the intracellular survival of Mtb.

### Mechanism of Lipid Accumulation of Foamy Macrophages by Mtb Infection and Their Implications for HDT

Recently, it has been reported that several bacterial factors of Mtb have additive modulatory effects on the lipid metabolism of host cells (Figure 1B). For example, ESAT-6 stimulates the translocation of glucose transporter GLUT-1, resulting in the active transport of glucose with the perturbation of metabolic flux (69, 70). Enhanced glucose metabolism leads to increased *de novo* lipid synthesis, which elevates the accumulation of lipids in foamy macrophages (69). It has been reported that activation of the G protein-coupled receptor GPR109A, an anti-lypolytic receptor, by ESAT-6 leads to the accumulation of lipid bodies in foamy macrophages, contributing to Mtb survival (70). In contrast, blockage of glycolysis with 2-deoxyglucose is detrimental to defense against Mtb infection because it disturbs M1-like activation in interstitial macrophages (39). Overall, glycolysis has contradictory roles against Mtb infection. By inducing pro-inflammatory immune responses, glycolysis with a broken TCA cycles supports anti-microbial responses in macrophages (39). However, excessive glycolysis is associated with elevated lipid accumulation by bacterial factors, including ESAT-6, to generate a niche that is suitable for Mtb (69, 70). Therefore, metabolic links between excessive glycolysis and lipogenesis are potential targets for reducing bacterial burdens with effective immune responses (Table 1).

Mtb also modulates nuclear transcription receptors of host cells involved in metabolic reprogramming and immune responses (71, 72). The Mtb-induced PPAR-γ pathway, which is a prominent signaling pathway that induces the activation of M2 macrophages, leads lipid accumulation to support the intracellular survival of Mtb. In this processes, PPAR-γ, with testicular receptor 4, increases the level of CD36 to contribute to lipid uptake (71). Another PPAR family member, PPAR-α, is a transcriptional inducer of FAO that prevents lipid accumulation in Mtb-infected foamy macrophages and promotes autophagy with transcription factor EB to reduce intracellular Mtb growth (73). Liver X-receptor (LXR)-α and LXR-β are also critical regulators of oxysterol metabolism that modulate immune responses in Mtb infection (74, 75). In mice with Mtb infection, LXR-α and LXR-β signaling is required to generate protective T cell responses (74). It has been reported that LXR signaling plays a role in regulating antimicrobial peptide expression to restrict Mtb growth via IL-36 (75).

In addition to modulating nuclear transcription factor signaling pathways, infection by Mtb induces tumor necrosis factor receptor signaling, followed by the activation of its downstream mTOR complex 1 (mTORC1) and caspase pathways in human primary macrophages (10). Activation of mTORC1 results in the accumulation of triglycerides in Mtb-infected macrophages. Mtb also induces miR-33 expression in foamy macrophages to inhibit lipid catabolism and autophagy, enabling intracellular survival and persistence (76).

Additionally, accumulating evidence has suggested that the disruption of lipid homeostasis, including hypercholesterolemia, leads to increased TB susceptibility. It is reported that apolipoprotein E-deficient mice have an increased susceptibility to hypercholesterolemia, showing defective priming of IFN-γ responses (77). Oxidized low-density lipoproteins, which are modified under hyperlipidemic conditions, also contribute to enhancing TB susceptibility via lysosomal dysfunction, impairing the control of Mtb survival (78). Statins, the 3-hydroxy-3-methylglutaryl-CoA reductase inhibitors for reducing cholesterol synthesis, have been attempted to treat Mtb infection (79–82). Clinically, treatment with statins has been reported to elicit improved outcomes for patients with TB (82, 83), but showed no additional effects in some cases (80, 81). By considering the characteristics of each patient, it is necessary for novel HDT approaches to modulate the immune response and/or cholesterol metabolism to be effective against TB. Collectively, these observations suggest that lipid metabolism and/or homeostatic pathways are promising targets for HDT against TB (Table 1, lower).

## Future Direction and Perspectives

To discover new therapeutic strategies against TB, many researches have attempted to alleviate Mtb infection through HDT. Foamy macrophages provide a niche for bacilli survival, maintenance, and persistence using their enriched cytosolic lipids. Even worse, necrosis of Mtb-infected foamy macrophages results in the generation of the necrotic core of TB granulomas, causing extracellular growth of Mtb. As Mtb actively utilizes lipids to generate energy and virulent factors from host cells, the regulation of host lipid metabolism pathways is a potential therapeutic strategy for alleviating TB by preventing vicious cycles between Mtb and macrophages.

In summary, alveolar and interstitial macrophages exert opposite roles against Mtb infection; therefore, understanding these macrophages is important for establishing strategies against TB pathogenesis. In TB pathogenesis, alveolar macrophages are susceptible to Mtb with elevated fatty acid uptake and FAO. In contrast, interstitial macrophages show resistance features like M1 macrophages in the early stage of Mtb infection. Pro-inflammatory M1 macrophages exhibit massively increased glycolysis and fatty acid synthesis with bactericidal activities. M2 macrophages show elevated lipid catabolism with suppressive activities in their antibacterial responses against Mtb. Thus, the Mtb-induced transformation processes from M1 to M2 macrophages are potential immunological targets of TB. Some recent revolutionary investigations have been conducted using *in vitro* granuloma and organoid culture systems to gain further insights into TB (42, 84–86). Using *in vitro* granuloma culture systems, M1 macrophages were shown to transform into M2 macrophages following Mtb infection (42). Although these techniques have only provided limited information of Mtb-infected macrophages to date, they have potential for further detailed investigations of Mtb-infected foamy macrophages to suggest more promising targets of HDT. Moreover, the metabolic pathways of Mtb-infected foamy macrophages perturbed by Mtb infection are important targets of HDT against TB. A recent study indicated that Mtb-infected foamy macrophages could uptake more fluoroquinolones compared to non-foamy macrophages *in vivo* (87), suggesting that it is important to consider the functions of foamy macrophages for anti-TB drug treatment. With consideration of the physiology of macrophages, it is expected that HDT strategy targeting immunological functions and/or metabolic perturbation of Mtb-infected foamy macrophages can be developed.

## Author Contributions

DS, HK, and SS wrote and revised the manuscript. SS conceptualized and edited the manuscript. All authors read and gave final approval of the manuscript.

## Conflict of Interest

The authors declare that the research was conducted in the absence of any commercial or financial relationships that could be construed as a potential conflict of interest.
