# Corrigendum: *Mycobacterium tuberculosis* Infection-Driven Foamy Macrophages and Their Implications in Tuberculosis Control as Targets for Host-Directed Therapy

**DOI:** 10.3389/fimmu.2020.01601

**Published:** 2020-07-17

**Authors:** Dahee Shim, Hagyu Kim, Sung Jae Shin

**Affiliations:** ^1^Department of Microbiology, Institute for Immunology and Immunological Diseases, Brain Korea 21 Program for Leading Universities and Students (PLUS) Project for Medical Science, Yonsei University College of Medicine, Seoul, South Korea; ^2^Department of Life Science, Research Institute for Natural Sciences, College of Natural Sciences, Hanyang University, Seoul, South Korea

**Keywords:** *Mycobacterium tuberculosis*, foamy macrophage, tuberculosis, immune responses, lipid metabolism, lung inflammation, host-directed therapy

In the original article, incorrect information and references were included in ***Interaction*** ***Between Mtb and Mtb–Driven Foamy Macrophages***, sub-section ***Characteristics of*** ***Mtb-infected Foamy Macrophages and the Utilization of Their Lipids by Mtb***, **Paragraph 3**:

A reference in the sentence “Furthermore, it has been suggested the use of lipids in host cells is related to dormancy of Mtb.” was incorrectly added as “Jaisinghani N, Dawa S, Singh K, Nandy A, Menon D, Bhandari PD, et al. Necrosis driven triglyceride synthesis primes macrophages for inflammation during *Mycobacterium tuberculosis* infection. *Front Immunol*. (2018) 9:1490. doi: 10.3389/fimmu.2018.01490.” This should be deleted.

In addition, there was a further error within the same paragraph:

“The region of difference 1 protein in Mtb contributes to increasing the levels of intracellular triglycerides in Mtb by enhancing the expression of diacylglycerol *O*-acyltransferase, a key enzyme in triglyceride synthesis (68).”

The text should be substituted with the following:

“The region of difference 1 in pathogenic mycobacteria may contribute to the interaction between diacylglycerol *O*-acyltransferase, a key enzyme in triglyceride synthesis, and lipid droplets of mycobacteria to generate intracellular lipid inclusions.” This statement is supported by the reference “Barisch C, Soldati T. *Mycobacterium marinum* degrades both triacylglycerols and phospholipids from its dictyostelium host to synthesise its own triacylglycerols and generate lipid inclusions. *PLoS Pathog*. (2017) 13:e1006095. doi: 10.1371/journal.ppat.1006095”.

The authors apologize for these errors and state that the revisions do not change the scientific conclusions of the article in any way. The original article has been updated.
